# Inclusion of learners with learning disabilities in the Vaal Triangle mainstream classrooms

**DOI:** 10.4102/ajod.v12i0.1163

**Published:** 2023-06-12

**Authors:** Nilford Hove, Nareadi T. Phasha

**Affiliations:** 1 Department of Education, Faculty of Inclusive Education, University of South Africa, Pretoria, South Africa

**Keywords:** inclusive education, mainstream classrooms, learners with learning disabilities, multi-level teaching, differentiated instruction, parental involvement, code switching

## Abstract

**Background:**

South Africa adopted a policy on inclusive education in 2001 to ensure that all learners are accommodated and accepted in the classrooms despite their differences.

**Objectives:**

This study was aimed at exploring the inclusion of learners with learning disabilities in mainstream primary schools for teaching and learning.

**Method:**

This study followed a qualitative approach embedded in a descriptive phenomenological design. Data were generated through in-depth interviews with individual participants and were analysed thematically for content. Six teachers from six different mainstream primary school classrooms were purposefully selected for the study.

**Results:**

Findings revealed that overcrowding, time constraints and lack of parental involvement impede the inclusion of learners with learning disabilities in mainstream classrooms. However, teachers use: (1) multi-level teaching, (2) concrete teaching and/or learning aids, (3) differentiated instruction and (4) code-switching in accommodating learners with learning disabilities.

**Conclusion:**

This study argues that for learners with learning disabilities to be more included in mainstream classrooms, the learner population should be reduced to a maximum of 30 learners per class, and collaboration with parents should be enhanced. Also, the arrangement of learners for teaching and learning could be limited to small groups consisting of four to five learners. Multi-level teaching and differentiated instruction should be applied in settings that do not require learners to be separated from their peers without learning disabilities.

**Contribution:**

This study will help improve teachers’ inclusive classroom pedagogical practices for all learners including those with learning disabilities.

## Introduction

Classrooms nowadays accommodate learners who are diverse in ability, age, race, background and all other aspects that make them different because of policy on inclusivity adopted in 1994 by many nations globally (Bubpha 2014). At the heart of inclusive education is a commitment to seeing learners of all kinds receiving education in the same spaces for their social and educational growth (UNESCO 2005). South African schools have seen persistent growth in the enrolment of learners who are diverse in mainstream classrooms since 2001, including learners with specific learning disabilities as a result of the adoption of policy on inclusion (Engelbrecht et al. 2015).

The Diagnostic and Statistical Manual of Mental Disorders Fifth Edition, Test Revision (DSM-V-TR) (American Psychiatric Association 2022) defines specific learning disability as a type of neurodevelopmental disorder that hinders an ability to learn or use specific academic skills (reading, writing or arithmetic), which are the foundation for other academic learning. Learners with a specific learning disability experience a greater difficulty in learning than the majority of their peers (Bryant, Bryant & Smith 2017). Specific learning disability is understood in terms of poor academic performance as compared to a difficulty posed because of physical, intellectual or sensory deficits. As a consequence of what these learners experience in their learning, they require attention in terms of curriculum adaptation, teaching methods, and additional or specialised teaching and learning materials among other needs (Udoba 2014).

Prior to the adoption of inclusive education in South Africa, Gwala-Ogisi in Phasha (2010:165) noted that learners with disabilities were placed in special programmes and remedial education programmes and further divided according to the severity of their disabilities. For example, those with slight specific learning disabilities received remedial assistance within mainstream classrooms, those with moderate specific learning disabilities were placed in temporary to full-time remedial services and those with severe learning disabilities were placed in special schools. Education practices followed a strict streaming system that differentiated between general, vocational and academic education based on learners’ abilities within schools (Dunne 2010). However, in light of the policy on inclusion that is currently in place, the special needs and the support that learners with learning disabilities require in the classrooms have to be received within the mainstream education system to facilitate their effective education for better academic and social developments (Florian 2015; UNESCO 1994).

Benefits of mainstream class teaching for learners with learning disabilities include positive teacher–student rapport, real-life connections and good use of strategies and modifications (Ford 2013). Other benefits of mainstream class teaching for learners with learning disabilities include increased participation by all learners in the learning process and decreased exclusion of those with learning disabilities in the curricula of mainstream schools (Florian 2015). Thus, classroom teaching practices are obliged to shift away from experiences that work for most learners, towards those that foster the development of rich learning environments, distinct with learning opportunities that are sufficiently made available for everyone so that all learners are able to participate in classroom life (Florian & Black-Hawkins 2011). Although there is a considerable amount of research on the inclusion of learners with learning disabilities elsewhere, there is a dearth of literature in the Vaal Triangle on how learners with learning disabilities are supported in mainstream classrooms.

### Literature review

Studies carried out globally on inclusive pedagogic practices in teaching learners with learning disabilities in mainstream classrooms pointed to challenges with the way these learners receive their education. Consequently, these challenges induce high stress levels in teachers as they try to include learners with learning disabilities in the daily classroom learning activities. For example, a study by Dick (2010) revealed that some teachers complain that teaching learners with learning disabilities while attending to other learners without learning disabilities is a stressful job, especially in instances where teachers are required to provide individualised instruction. This is compounded by their inability to differentiate instruction when teaching learners who are widely diverse (Hashir 2018). In other instances, the challenges are worsened where learners have to learn or study through a medium of instruction other than their own mother tongue. Additionally, a study by Mackey (2014) in the United States of America established that teachers felt they had not been sufficiently prepared to teach learners with learning disabilities as they had studied only one undergraduate special education course.

In other cases, teachers show confusion regarding the provision of support to learners with learning disabilities because they are unaware of the law pertaining to assessments and supports for learners with special educational needs (Ford 2013). For example, a qualitative study by Mntambo (2011) in Lesotho that explored teachers’ experiences in teaching learners with learning disabilities revealed a lack of understanding of inclusive education practices by the teachers. Instead, teachers prefer to focus more on visual and physical impairments than on learners with learning disabilities.

The availability of teaching and/or learning materials that address the specific needs of individual learners is an important prerequisite for meaningful teaching and learning for those with learning disabilities. The shortage of such materials has negative consequences on the inclusion of learners with learning disabilities, potentially complicating teaching and learning. A study by Udoba (2014) in Tanzanian mainstream primary schools revealed that teachers cease to give focussed attention to learners with learning disabilities because of a shortage of teaching and/or learning materials like textbooks. Instead, teaching is more focussed on those who are average and those whose academic performances are above average. Teachers struggle with learners who lack basic skills in reading, writing and mathematics as they attempt to make them realise their full potential (Udoba 2014). A similar finding was reported in a study conducted by Ngonyani (2010) in Tanzania, which noted that the problem is further compounded by the shortage of reading materials and books giving the teacher the extra task of writing the learning notes every day. Other contributing factors included a lack of guidelines that direct teaching of all learners in the same classrooms.

Some teachers complain that learners with learning disabilities are at risk of not learning under general education classrooms for long-term academic achievements, as well as for their social benefits because the classrooms are overcrowded (Garnett 2022). Classroom instructions in overcrowded classrooms tend to be directed at large groups of learners, focussing more on what has to be learnt and not necessarily on the levels of the learners. Florian and Black-Hawkins (2011) noted that in England, there are some fundamental constraints that exist within education systems and across schools that counter teachers’ efforts to be more inclusive in their practices. For example, the measuring of learners’ academic performance at ages 7, 11, 14 and 16 years through standardised tests and the publication of the results influence teachers to focus more on learners who attain good results than those who are struggling academically (Florian 2015).

Despite the challenges that teachers face in teaching learners with learning disabilities in mainstream classrooms, a qualitative study by Morton (2007) in South Western Austria exploring teachers’ experiences in teaching learners with learning disabilities revealed that some teachers express feelings of loyalty and attachment to their learners with learning disabilities when they have produced positive results. From the same study, teachers reported that they often utilised group work and paired learners with learning disabilities with those without learning disabilities to facilitate their learning. Equally, a study by Maciver et al. (2018) in Edinburg revealed that teachers who have experience in working with students with special educational needs feel more positive towards learners with learning disabilities. In addition, Morton (2007) posited that primary school teachers of learners with learning disabilities work very hard to provide their learners with high-quality level of education. Furthermore, a study by Education Review Office (ERO) (2015) in New Zealand revealed that teachers encouraged all learners to accept diversity through working together with peers in small groups. Other studies indicate that teachers in mainstream classrooms are aware of the presence of learners with learning disabilities in their classrooms, and they make special arrangements to accommodate these learners so that they can benefit from the teaching and learning. For example, studies by Mwajabu and Milinga (2017) in Tanzania indicated that teachers consider learners’ disabilities and arrange the seating of learners according to their needs.

South African studies highlight pertinent glitches related to the inclusion of learners with learning disabilities in mainstream classrooms. An investigation by Lessing (2010) in 350 South African schools revealed that teachers lack the confidence to support learners with learning disabilities to overcome the barriers that they experience in their various developmental skills. Teachers are frustrated because they are unable to handle challenges faced by learners with learning disabilities as consequences of external forces like abuse at home (Mahlo 2011). Further studies reveal that teachers feel that although they have received some basic training in inclusive education, they still lack the basic skills like curriculum differentiation, which helps in assisting learners with learning disabilities. In line with the above, teachers in South Africa indicate that they need intensive training in inclusive education so that they are able to support learners with learning disabilities in their classrooms (Mahlo 2011).

A qualitative study by Bojuwoye et al. (2014) in selected Western Cape schools in South Africa exploring learners’ experiences regarding the provision of support services revealed that learners with learning disabilities are grouped together in order to receive extra classes from teachers. Ngcobo and Muthukrishna’s (2011) study that explored a school-based initiative including children with disabilities in KwaZulu-Natal revealed that there is some form of hierarchy in the classrooms where learners are arranged based on their abilities. However, such practices are rejected by Florian and Black-Hawkins (2011) who argue that assisting some learners separately leads to labelling of some learners who would have been identified and grouped alone because they have learning disabilities.

In addition to the above, a qualitative study by Zwane and Malale (2018) in the Gege branch of Swaziland revealed a lack of facilities in government schools and teachers’ incompetency in identifying learners facing learning challenges. A study conducted in Gauteng by Yoro, Fourie and Van Der Merwe (2020), which captured perspectives of recently qualified teachers about the learning support strategies for learners with neurodevelopmental disorders, revealed that teachers use a variety of strategies such as cooperative learning, peer learning, ability grouping visual aids and curriculum differentiation. However, they noted that those strategies tend to be general, and they noted a need for teachers to use more support strategies in regular classrooms. Although this study sheds light on the situation in schools located in Gauteng province, it cannot be generalised to other parts of the province. Vaal Triangle in particular, an industrial city located in the southern parts of Gauteng province, is highly diverse in terms of its population with a wide range of migrants.

### Theoretical framework

This study was carried out through the lens of inclusive pedagogy by Florian and Black-Hawkins’ (2011). The theory stresses that all learning in the classrooms should take place within the same rich environments that have been carefully crafted to meet the needs of all learners despite their differences. Fundamentally, inclusive pedagogy emphasises a change in teaching and learning approaches from those that work for some learners towards those that involve learning opportunities that suit all learners (Florian & Black-Hawkins 2011). Teachers need to respect and respond to learners’ differences and be able to include everyone rather than excluding some from what is generally available in their classrooms (Florian 2007).

Background to the study has revealed that learners with learning disabilities now form part of the learner populations in mainstream classrooms because of the policy on inclusion. As such, these learners need to be taught and learn in ways that embrace their challenges, as well as give them support when they need it, and ensure improvement in their academic attainments without being excluded from any classroom arrangements which might compromise their educational progress.

### Objectives of the study

This study was aimed at exploring the inclusion of learners with learning disabilities in the Vaal Triangle mainstream primary schools for teaching and learning, through answering to the question: How are learners with learning disabilities included in mainstream classrooms for teaching and learning?

## Research methods and design

A qualitative approach embedded in a descriptive phenomenology design was used in this study. The approach is based on the collection of verbal data that are often presented in narrative accounts (Arthur et al. 2012). The qualitative research approach offers the researcher the benefit of understanding social phenomena from participants’ perspectives, using their own voices. In the same vein, the phenomenological design enables a researcher to uncover what several participants who experience a phenomenon have in common (Creswell 2013). Perspectives include participants’ feelings, beliefs, ideas and thoughts regarding the phenomena under study. Furthermore, phenomenologists seek to understand how participants experience the phenomena under study. In the context of this study, the approach allowed the authors the opportunity to explore the participants’ experiences in depth and allowed for a nuanced understanding of their daily experiences in teaching learners with learning disabilities in mainstream classrooms. In that regard, a deeper understanding of classroom pedagogic practices in teaching learners with learning disabilities, together with all others without learning disabilities was obtained. As educators with special and inclusive education background, it was imperative that the authors put aside their beliefs and knowledge about the phenomenon studied. The authors adhered to the principle of bracketing by involving an independent researcher to check the research questions that guided the interview, the audio recording as well as the transcripts. The researches kept a journal in which they recorded their beliefs about some issues that emerged from the interviews and used it during data analysis. The journal was made available to the independent researcher as he checked the analysed data, especially the themes that the authors developed.

### Participants and setting

This study was undertaken in the Vaal Triangle, one of the Department of Education Districts in the Johannesburg region of South Africa. Schools in this district are categorised as non-fee-paying, as they accommodate learners from communities with low–income households. Furthermore, Vaal Triangle is an area that accommodates diverse learners from surrounding farms, as well as from peri-urban and urban settings. Six educators from six different schools were purposefully selected to take part in this study. The power and logic of purposive sampling are that a few cases studied in depth yield many insights about the topic (McMillan & Schumacher 2012). In purposeful sampling, participants are chosen because they are likely to be knowledgeable and richly informative about the phenomena the researcher is investigating (McMillan & Schumacher 2012). In the same vein, the logic and power of purposeful sampling lie in selecting information-rich cases for study in depth (Lodico, Spaulding & Voegtle 2010). Information-rich cases are those from which one can learn a great deal about issues of central importance to the purpose of the study.

Particular criteria were set in order to identify and select the participants. The criteria that were set which participants were expected to meet related to: (1) understanding of South African policies on Inclusive Education and (2) more than 5 years’ experience teaching learners with learning disabilities in mainstream classrooms. The authors received assistance from the District-Based Support Team in identifying the schools that had participants who met the above criteria, after which they sought permission from principals of the identified schools to have access to the participants. The School-Based Support Teams assisted in identifying teachers who met the set criteria and were deemed to be the most information-rich cases. The purpose of the study was explained to the identified participants and they were informed that participation was voluntary.

### Data collection

Data were collected using in-depth interviews. Interviews lead to face-to-face engagements with research participants which permit probing deeply into participants’ experiences (Atkins & Wallace 2012). Interviews are also ideal when the researcher wishes to follow up initial responses by probing for additional information that can clarify existing data (Savin-Baden & Major 2013). The researchers carried out in-depth interviews with participants on how they teach learners with learning disabilities in mainstream classrooms. All the interviews were carried out in English as a standard medium of communication. However, participants were encouraged to express themselves in vernacular where they were facing difficulties in expressing themselves, after which the authors translated their views into English with the help of other teachers who spoke the same language. The authors held two separate 60 min interviews with each participant in the afternoons at the places where they work. The two interviews with each participant were spaced between 2 weeks from each other in order to get more information and clarity on what they had said in the first interviews. The interviews took place after school hours, and they were audio recorded with the participants’ permission.

### Data analysis

Data were analysed thematically using a model by Lodico et al. (2010), which has six key steps, namely: (1) preparation and organisation of data; (2) reviewing and exploring the data; (3) coding data into categories; (4) conducting thick descriptions of people, places and activities; (5) building themes; and (6) data reporting and interpretation. The analysis began with data preparation and organisation of the collected data, which entail putting it in a form that can easily be analysed. Data transcription that was undertaken related to the following: (1) site or location from which data were collected, and (2) persons studied. The authors read through the data looking at the various types of data collected and wrote down words and phrases that captured the important aspects of the data. Data were then coded into categories according to how they were related in describing certain aspects. The idea was to put related data together for easy discussion and interpretation.

### Ethical considerations

Ethical clearance was obtained from the University of South Africa College of Education Research Ethics Review Committee (No. 2015/05/13/47000872/ 22/MC). This indicates that the study met basic ethical standards. The ethical clearance certificate was obtained before data collection commenced. Participants with assistance from the District-Based Support Team and School-Based Support Teams from the selected schools were identified. The participants were given a full description of the study, what it aimed to achieve, including the methods to be used. They were also made aware that participation in the study was voluntary, and they can withdraw at any stage of the research process without negative consequences. They signed consent forms that explained what it meant to be involved in the study, and that they could make an informed choice to participate in the study or not to. The principle of participation without payment was clarified, and they were informed that interviews will take place in the afternoons at their place of work places to avoid interference with their work activities. The authors also assured the participants that what they would say would not be disclosed to other people other than the researchers, and neither would such information be traceable back to them as their real names would not be used in the final reporting of the findings. This was in alignment with the principle of confidentiality and anonymity. Participants were also asked to consent to audio-recording as that was important for the accurate collection of data. The authors promised that they will be afforded an opportunity to verify analysed data. More details regarding the participants are displayed in Table 1.

**TABLE 1:** Participant information.

Participant	Age (years)	Gender	Occupation	Experience (years)	Qualification
A	45	Male	Teacher	16	Diploma in Education, a Bachelor of Science in Special Needs Education; and Honours degree in Inclusive Education
B	46	Male	Teacher	18	Certificate in Education; and Bachelor of Education degree in Special Needs Education
C	38	Female	Teacher	12	Diploma in Education; and Advanced Certificate in Education specialising in Inclusive Education
D	49	Female	Teacher	19	Diploma in Education; Bachelor of Education in Special Needs Education; and Honours degree in Inclusive Education
E	40	Female	Teacher	19	Diploma in Education, an Advanced Certificate in Special Needs Education; and Bachelor of Education in Special Needs Education
F	43	Female	Teacher	22	Diploma in Education, an Advanced Certificate in Special Needs Education; and Bachelor of Education in Special Needs Education

## Findings and discussions

Results of this study revealed that although learners with learning disabilities are being included in mainstream classrooms for teaching and learning, there are some deep-rooted challenges that negatively impact teachers’ efforts to give maximum attention to all learners, including those with learning disabilities. These challenges relate to overcrowding in the classrooms, time constraints in giving focussed attention to individual learners and a lack of parental involvement in the education of their children with learning disabilities.

Four participants indicated that even though they are aware of the need to effectively teach learners with learning disabilities alongside others, overcrowding in the classrooms is a huge hindrance to inclusive classroom practices, as evidenced by participant E who was quoted saying:

‘It’s not practical to reach out to all learners due to learner population. We try to attend to all learners, especially in concepts that you know you can’t teach in the same way, you cannot use one approach, if you do, some learners will miss out. However, the numbers of learners per class are just too high to give individual attention to some learners.’ (Participant E)

Equally, participant C indicated that although they practice multi-level teaching in the classrooms, overcrowding erodes some of their efforts as she stated that:

‘We do multi-level teaching, but it’s hard to do multi-level teaching because we have quite a large number of learners in the classes. There are many learners in the classes. If they were two, I can, but honestly how can I support 15 learners [*learners with learning disabilities*] in one class. I will not be as effective the way I would want.’ (Participant C)

Overcrowding emerged as one of the major drawbacks to the inclusion of learners with learning disabilities in mainstream classrooms. The study’s findings revealed that class sizes in mainstream classrooms are generally too high, with as many as 50 learners per class. A similar finding in Botswana by Otukile-Mongwaketse’s (2018) study established that large class sizes impede on principles of inclusivity in Botswana. Arguably, environments in which learners with learning disabilities receive instructional services affect how they learn, as well as the quality of education they receive (Heward 2014). Such learning environments may not be manageable, and teachers may not be able to give each learner the adequate attention they need to grasp learning content. Maximising participation of learners with learning disabilities in the curriculum of the school can be achieved through optimising opportunities for learner engagements in mainstream classes, which, however, are being hampered by the large class sizes in the regular classrooms as indicated in the findings of this study. Conversely, smaller class sizes can afford teachers the opportunity to cope with the added responsibility of teaching widely diverse learners in the regular classes (Lerner & Johns 2012).

All six participants complained about a lack of time to effectively include learners with learning disabilities in the classrooms, as shown by participant F who explained:

‘It’s time consuming [*giving individual support to learners with learning disabilities*]. There are a lot of them who are struggling. As a teacher you see that your expertise is needed, but it’s time consuming to prepare work specifically for them.’ (Participant F)

Participant B bemoaned the amount of paperwork they have to work on which negatively impacts on teachers’ ability to reach out to those with learning disabilities as they need more help. He argued that:

‘We have a lot of admin work as teachers. We don’t spend enough time focussing on our work because we have to do these stats from the District. This makes it very hard for me to have time to help these learners who need more assistance.’ (Participant B)

The above was corroborated by participant C who stated that:

‘The challenge is the time and space. We are a normal school. We are expected to submit or to produce, to meet passing requirements as a normal school. As teachers we are much aware about what learners are facing and what should be done, but the environment and the space do not allow us time to do what we should be doing in the classrooms.’ (Participant C)

Results indicate that teachers find it highly difficult to attend to learners with learning disabilities in mainstream classrooms because of lack of time as a result of commitments to other classroom obligations. In Botswana, teachers end up teaching learners with learning disabilities as part of one large group, treating them as passive recipients of knowledge and depriving them of the opportunity to receive extra attention and support from the teacher (Nkobi 2011; Otukile-Mongwaketse 2018). In Tanzania, teachers focus more on learners who put their hands up and leaving out those with learning disabilities because of lack of adequate time to attend to all learners (Miles, Westbrook & Croft 2018). However, in the light of inclusive pedagogy, teachers should be able to respect and respond to learner needs in ways that include all, rather than exclude some from what is ordinarily available in the daily life of the classroom (Florian & Black-Hawkins 2011). Arguably, smaller class sizes will reduce the burden on teachers, enabling them to attend to the needs of those with learning disabilities.

A lack of parental involvement in the education of their children also poses a threat to the inclusion of learners with learning disabilities. Findings revealed that parents’ lack of cooperation when learners have to be sent for further assessments and other psychological services frustrates teachers. This was pronounced by participant C who was captured saying:

‘In my class there is one learner who has ADHD, and another one with Down’s syndrome. Both of them can’t read fluently, they are struggling, but the parents don’t want to take their children for assessments. There is not much that we can do as teachers without information and recommendations from psychologists, especially with an incident that involve ADHD.’ (Participant C)

She added that:

‘The children with ADHD can’t read … they can’t write, they are busy playing. Sometimes they use their pencils to poke other children. The parent does not want to give consent for assessment. We are struggling with parents who are not cooperating.’ (Participant C)

In the same vein, participant A stated that:

‘We have these parents of learners with learning disabilities. They don’t want to come for meetings. When they come for meetings, they are in denial about their children’s level of performance.’ (Participant A)

Including learners with learning disabilities goes beyond what teachers do in the classrooms. It also encompasses what parents are doing, or not doing, to facilitate learning and the inclusion of their children in mainstream classrooms. In that light, parental involvement is key to the educational growth of children with special educational needs, including those with learning disabilities (UNESCO 2005). Increased parental involvement is considerably linked to learner’s increased academic achievements (Topr et al. 2010).

Similarly, Afolabi, Sourav and Nenty (2013) asserted that parents’ involvement in the education of their children is a very important ingredient for successful inclusive practice. Parents are social actors in the education of their children whose roles include networking with teachers on issues that affect the child’s learning and giving consent for psychological assessments that their children may need. As revealed by this study, lack of parental involvement in instances such as psychological assessments, or cooperation with teachers at meetings deprive learners with learning disabilities of very important supports for inclusion as envisaged in the Department of Education (2014).

In spite of the above drawbacks faced by teachers in trying to be more inclusive towards learners with learning disabilities, results of this study revealed that teachers use different teaching and/or learning approaches in mainstream classrooms. These different approaches include multi-level teaching, use of concrete teaching and/or learning aids, curriculum differentiation and code-switching.

Four participants indicated that they use multi-level teaching that entails scaffolding content starting from simple to more complex activities in order to accommodate learners with learning disabilities, as evidenced by participant C who stated that:

‘What we do is we use multi-level teaching. We teach them starting from the simplest concepts moving towards the most difficult so that all learners can understand. You can’t teach them [*learners*] in the same way because they are different. You find that you have someone with ADHD in class, someone who cannot spell, and someone who is able to do everything in the same class.’ (Participant C)

The above was corroborated by participant E who explained that:

‘Learners are not the same in achievements, so I try to use multi-level teaching in my class. Here I try to meet the needs of all the learners by starting from the simplest to the more complex tasks. This enables all learners to at least achieve something from what they are learning.’ (Participant E)

Results indicate that teachers use multi-level teaching approaches in the classrooms to enable all learners to master concepts starting from what is generally achievable by all learners, moving towards more difficult tasks. Similar findings have been established by Benmassoud and Madani (2019) in a study in Morocco, which revealed that multi-level teaching approaches are common practices in the classrooms that afford brighter learners to benefit from demonstrating some skills to weaker learners, while those who are weak can learn a great deal from their counterparts without learning disabilities. Arguably, this is in line with one of the inclusive education goals of ensuring that all learners benefit from the curriculum of the school (Florian 2015). Although it may not be possible for teachers to serve every learner’s needs when using multi-level teaching, especially in classrooms that have high numbers of learners (Lynch 2022), the practice augurs well with principles of inclusive education in that teaching and learning takes place in the same spaces for all learners without leaving out others (Florian & Black-Hawkins 2011).

On the contrary, three participants indicated that they facilitate effective learning for those with learning disabilities in mainstream classrooms through the use of concrete teaching and/or learning aids, as evidenced by participate A who was captured saying:

‘I use teaching aids. I give them things to touch, like counters. Those who are struggling can learn better through the use of concrete aids, at times charts or pictures. I use them in my classes so that all learners can benefit. They need these things.’ (Participant A)

Similarly, participant D asserted that:

‘I try to do that justice for certain concepts like when you are doing counting. I use counters and show them pictures so that they can understand and feel catered for.’ (Participant D)

The use of teaching and/or learning aids can increase, maintain or improve the functional capabilities of learners with special educational needs, like those with learning disabilities (Vaughn, Bos & Schumm 2011). In the same vein, Heward (2014) argued that learners with learning disabilities can improve their comprehension through the use of graphic organisers and other visual representations of elements of narrative stories. The above is further supported by Smith et al. (2011) who posited that providing a wide range of concrete teaching and/or learning aids can encourage success in writing for learners who are struggling with shaping letters or numbers. Rich environments should be created within the learning centres for learners of different abilities, especially for those with learning disabilities to learn through manipulation (Department of Education 2014). Arguably, rich learning environments can be made available through the provision of concrete teaching and/or learning aids, which is in alignment with one of the principles of inclusive education that calls for the need to avail various teaching and/or learning aids to learners with learning disabilities in order to maximise learning. However, in the light of inclusive pedagogy, the provision of teaching and/or learning aids should be extended from what is ordinarily available to all learners in the class, rather than making ‘different’ or ‘additional’ provision for some individuals who might be experiencing difficulties in their learning (Florian & Black-Hawkins 2011).

Differentiated instruction emerged as one of the approaches teachers use in the classrooms in order to accommodate learners with learning disabilities. Differentiating instruction entails asking questions differently according to learners’ levels of understanding, as well as giving simpler tasks on the same concept to learners who are struggling, as was stated by participant B who was captured saying:

‘We do curriculum differentiation. There are learners who cannot write at all due to motor skills, and there are learners who cannot read due to other challenges maybe because of their foundation. However, we do curriculum differentiation whereby the same concept in grade 6, I will ask the same questions differently in a grade 6 level, we lower the content to meet that gap.’ (Participant B)

The above was reinforced by participant F who indicated that:

‘Some of them they cannot read. We give them simpler tasks different from what we give others, say at Grade 5 level. We don’t give them the same tasks but the concept is the same.’ (Participant F)

Teachers must account for individual learners’ differences when giving out instructional activities in the classrooms, which can be done through differentiating instructions (Frederickson & Cline 2011). However, learners must not be separated in order to receive differentiated instruction as such a practice embodies the qualities of clinical teaching, which is against the principles of inclusive education (Lerner & Johns 2012). In the same vein, Spratt and Florian (2013) argued that all learners can make progress within the same environment if conditions are right, essentially implying that they can still be given differentiated instruction without necessarily being separated from others (Florian & Black-Hawkins 2011).

Five participants indicated that they use code-switching in order to accommodate learners with learning disabilities in the classrooms, as evidenced by participant A who was captured saying:

‘I also use code switching. My learners are mostly Sotho speakers and Zulu speakers. We are allowed to, but not to over use code switching. I will switch to Sesotho or Isizulu to explain a concept especially in the beginning of an activity.’ (Participant A)

The above was supported by participant B who stated that:

‘At times I use vernacular or the language that the learner can understand. You see that the child is not getting what you are saying because I am teaching in English which is a second language to them. Once I see that, I try to explain the concept in a language that the learner speaks, that is if I am good at that language as well.’ (Participant B)

Classroom code-switching refers to the practice of using more than one linguistic code in the classrooms by both teachers and learners (Lin 2017). Findings indicate that teachers use code-switching in the classrooms to accommodate learners with learning disabilities, where the medium of instruction is their second language. Essentially, code-switching is used so that learners can understand some concepts better in their home language. In that vein, code-switching increases participation by all learners in the classrooms, and learners end up having a better understanding of English grammar rules even if it is not their home language (Simasiku 2016). In light of the policy of inclusion, it can be argued that code-switching increases participation by all learners in the classrooms (UNESCO 2005). Inadvertently, language can pose a major barrier to learning, especially if the medium of instruction is not the language learners speak at home.

### Recommendations and implications

Overcrowded classrooms undermine teachers’ efficacies to provide inclusive learning environments that are capable of facilitating meaningful teaching and learning for learners with learning disabilities in mainstream classrooms. At times, they fail to find time to attend to individual learners’ needs. To mitigate these challenges, a number of learners in the classrooms should be reduced to a maximum of 30 learners per class so that teachers will be able to attend to all learners. Collaboration with parents is important for the effective support of learners with learning disabilities. In addition, parents are more knowledgeable than any other adult about their children; therefore, their involvement in the education of their children could help the teacher understand the child better. Moreover, as noted by Swart and Phasha (2019), the responsibility for educating and socialising children should be a shared responsibility because the inclusion of learners with learning challenges requires both teachers and parents to assume different responsibilities.

The study’s findings also revealed that teachers’ use of strategies such as multi-level teaching and differentiated instruction in order to accommodate those with learning disabilities indicates their reception to inclusive practices. The maximum benefit of such methods could be felt if they are applied in settings that do not require them to be separated from their peers without learning challenges. Also, the arrangement of learners for teaching and learning could be limited to small groups consisting of four to five earners. Small groups could benefit them socially and academically. They are effective in facilitating member interaction and keeping learners with learning disabilities motivated to learn as they will get the attention they need. Their confidence could be boosted as they will feel comfortable to ask questions.

### Limitations

This study was carried out in the Vaal Triangle of South Africa, involving six mainstream classroom teachers at the primary school level. Schools selected for this study were non-fee-paying schools from poor communities. As such, the findings of this study may not be representative of other categories of schools that are not non-fee-paying. Essentially, the results of this study do not reflect on how learners with learning disabilities are included in mainstream classes in other settings that might be having different experiences regarding the inclusion of learners with learning disabilities. Furthermore, teachers selected for this study had professional qualifications in inclusive education or special needs education and more than 5 years’ teaching experience in mainstream primary schools. In that regard, the voices of participants in this study cannot be generalised to other practitioners of different characteristics.

## Conclusion

The study sought to investigate the inclusiveness of mainstream classroom instructional practices for learners with learning disabilities. A qualitative approach embedded in phenomenological design was used in this study. Six participants were purposefully selected following the given criteria. Findings revealed challenges that are related to the inclusion of learners with learning disabilities in mainstream classrooms, which include large class sizes, lack of time to attend to individual learners who need more support and lack of parental involvement in the education of their children. Further findings revealed that teachers use multi-level teaching, concrete teaching and/or learning aids, differentiated instructions and code-switching when teaching learners with learning disabilities in mainstream classrooms.

## References

[CIT0001] Afolabi, O.E., Sourav, M. & Nenty, H.J., 2013, ‘Implementation of inclusive education: Do parents really matter?’, *Specijalna Educakacija: Rehabilitacija* 12(3), 373–401. 10.5937/specedreh12-4370

[CIT0002] American Psychiatric Association, 2022, *Diagnostic and statistical manual of mental disorders*, 6th edn., American Psychological Association, Washington, DC.

[CIT0003] Arthur, J., Waring, M., Coe, R. & Hedges, L.V., 2012, *Research methods and methodologies in education*, Sage, Thousand Oaks, CA.

[CIT0004] Atkins, L. & Wallace, S., 2012, *Qualitative research in education*, Sage, Thousand Oaks, CA.

[CIT0005] Benmassoud, J. & Madani, E., 2019, ‘The teaching multilevel classrooms in Morocco. The case study of high schools in Meknes’, *GPH-International Journal of Educational Research* 2(3), 1–14.

[CIT0006] Bojuwoye, O., Moletsane, M., Stofile, S., Moolla, N. & Sylvester, F., 2014, ‘Learners’ experiences of learning support in selected Western Cape schools’, *South African Journal of Education* 34(1), 136–147. 10.15700/201412121002

[CIT0007] Bryant, D.P., Bryant, B.R. & Smith, D.D., 2017, ‘Teaching students with special needs in inclusive classrooms: Special educational needs’, *ELT Journal* 71(4), 525–528. 10.1093/elt/ccx042

[CIT0008] Bubpha, S., 2014, ‘Models of inclusive education: One size does not fit all’, *International Journal of Technology and Inclusive Education* 3(2), 328–334. 10.20533/ijtie.2047.0533.2014.0042

[CIT0009] Creswell, J.W., 2013, *Qualitative inquiry and research design: Choosing among five approaches*, 3rd edn., Sage, Thousand Oaks, CA.

[CIT0010] Department of Education, 2014, *Policy on screening, identification, assessment and support*, Government Printers, Pretoria.

[CIT0011] Dick, C., 2010, ‘How do they cope?: Teaching students with learning difficulties in mainstream classrooms’, Hons thesis, Faculty of Computing, Health and Science, Edith Cowan University.

[CIT0012] Dunne, A., 2010, *Dividing lines: Streaming access to the curriculum and junior certificate achievement in Ireland*, Combat Poverty Agency, Working Paper Series 10/05, Poverty Research Initiative, Dublin.

[CIT0013] Engelbrecht, P., Nel, M., Nel, N. & Tlale, D., 2015, ‘Enacting understanding of inclusion in complex contexts: Classroom practices of South African teachers’, *South African Journal of Education* 35(3), 1–10. 10.15700/saje.v35n3a1074

[CIT0014] ERO, 2015, *Inclusive practices for students with special needs in schools*, New Zealand Government, viewed 13 March 2022, from www.ero.govt.nz.

[CIT0015] Florian, L., 2007, ‘Reimagining special education’, in L. Florian (ed.), *The Sage handbook 634 of special education*, pp. 7–10, Sage, Thousand Oaks, CA.

[CIT0016] Florian, L., 2015, ‘Inclusive pedagogy: A transformative approach to individual differences but can it help reduce educational inequalities’, *Scottish Educational Review* 47(1), 5–14. 10.1163/27730840-04701003

[CIT0017] Florian, L. & Black-Hawkins, K., 2011, ‘Exploring inclusive pedagogy’, *British Educational Research Journal* 37(5), 813–828. 10.1080/01411926.2010.501096

[CIT0018] Ford, J., 2013, ‘Educating students with learning disabilities in inclusive classrooms’, *Electronic Journal for Inclusive Education* 3(1), 231–243.

[CIT0019] Frederickson, N. & Cline, T., 2011, *Special educational needs, inclusion and diversity*, Bell and Bain, Glasgow.

[CIT0020] Garnett, K., 2022, *What are classrooms like for students with learning disabilities?* viewed 10 November 2021, from https://www.readingrockets.org/article/what-are-classrooms-students-learning-disabilities.

[CIT0021] Hashir, A., 2018, *Challenges faced by teachers while teaching learners with learning disabilities*, viewed 13 October 2021, from https://www.researchgate.net/publication/325380717.

[CIT0022] Heward, L.W., 2014, *Exceptional children: An introduction to special education*, Pearson, Upper Saddle River, NJ.

[CIT0023] Lerner, W.J. & Johns, B., 2012, *Learning disabilities and related mild disabilities, characteristics, teaching strategies and new directions*, 12th edn., Cengage Learning, Wadsworth, OH.

[CIT0024] Lessing, A., 2010, ‘The ability of teachers to accommodate learners with learning disabilities in the inclusive classroom’, *Journal of Educational Studies* 9(2), viewed 13 January 2022, from https://hdl.handle.net/10520/EJC160151.

[CIT0025] Lin, A., 2017, *Code-switching in the classroom: Research paradigms and approaches*, viewed 03 March 2022, from https://www.researchgate.net/publication/318132410.

[CIT0026] Lodico, G.M., Spaulding, T.D. & Voegtle, K.H., 2010, *Methods in educational research: From theory to practice*, Jossey-Bass, San Francisco, CA.

[CIT0027] Lynch, M., 2022, *Inclusion vs. mainstreaming: What you need to know before putting your child in a classroom program*, viewed 23 May 2022, from https://www.theedadvocate.org/inclusion-vs….

[CIT0028] Maciver, D., Hunter, C., Adamson, A., Grayson, Z., Forsyth, K. & Mcleod, I., 2018, ‘Supporting successful inclusive practices for learners with disabilities in high schools: A multisite, mixed method collective case study’, *Disability and Rehabilitation* 40(14), 1708–1717. 10.1080/09638288.2017.1306586 28376644

[CIT0029] Mackey, M., 2014, ‘Inclusive education in the United States: Middle school general education teachers’ approaches to inclusion’, *International Journal of Instruction* 7(2), 5–20.

[CIT0030] Mahlo, F.D., 2011, *Experiences of learning support teachers in the foundation phase with reference to the implementation of inclusive education in Gauteng*, University of South Africa, Pretoria.

[CIT0031] McMillan, J.H. & Schumacher, S., 2012, *Evidence-based inquiry*, 7th edn., Pearson, Upper Saddle River, NJ.

[CIT0032] Miles, S., Westbrook, J. & Croft, A., 2018, ‘Inclusions and exclusions in rural Tanzanian primary schools: Material barriers, teacher agency and disability equity’, *Social Inclusion* 6(1), 73–81. 10.17645/si.v6i1.1203

[CIT0033] Mntambo, M.B., 2011, *An exploration of educators’ experiences in teaching learners with learning disabilities in three primary schools in Lesotho: Case study*, viewed 15 February 2022, from https://researchspace.ukzn.ac.za/xmlui/handle/10413/5816.

[CIT0034] Morton, A., 2007, *Teachers’ experiences with teaching children with learning difficulties: A qualitative study*, viewed 29 January 2022, from https://ro.ecu.edu.au/cgi/viewcontent.cgi?article=2109&context=theses_hons.

[CIT0035] Mwajabu, K.P. & Milinga, J.R., 2017, ‘Learner diversity in inclusive classrooms: The interplay of language of instruction, gender and disability’, *Malaysian Online Journal of Educational Sciences* 5(3), 28–45.

[CIT0036] Ngcobo, J. & Muthukrishna, N., 2011, ‘The geographies of inclusion of students with disabilities in an ordinary school’, *South African Journal of Education* 31(3), 358–369. 10.15700/saje.v31n3a541

[CIT0037] Ngonyani, M.S., 2010, ‘Teachers’ facilitation of learning for learners with disabilities in inclusive classrooms in Tanzania: Teachers’ use of interactive teaching methods in inclusive classrooms’, MPhil thesis, Universiteitet Oslo.

[CIT0038] Nkobi, O.P., 2011, ‘Silent exclusion: The unheard voices in remote areas of Botswana’, *International Journal of Educational Sciences* 3(2), 109–118. 10.1080/09751122.2011.11890015

[CIT0039] Otukile-Mongwaketse, M., 2018, ‘Teacher-centered dominated approaches: Their implications for today’s inclusive classrooms’, *International Journal of Psychology and Counselling* 10(2), 11–21. 10.5897/IJPC2016.0393

[CIT0040] Phasha, N., 2010, ‘Inclusive education in the South African context’, in E. Lemmer & N. Van Wyk (eds.), *Themes in South African education*, pp. 163–180, Pearson Education, Cape Town.

[CIT0041] Savin-Baden, M. & Major, H.C., 2013, *Qualitative research: The essential guide to theory and practice*, Routledge, London.

[CIT0042] Simasiku, L., 2016, ‘The impact of code switching in learners’ participation during classroom practice’, *Studies in English Language Teaching* 4(2), 157–166. 10.22158/selt.v4n2p157

[CIT0043] Smith, E.C.T., Polloway, A.E., Patton, R.J. & Dowdy, A.C., 2011, *Teaching students with special needs in inclusive settings*, pp. 269–290, Allyn and Bacon, Boston, MA.

[CIT0044] Spratt, J. & Florian, L., 2013, ‘Applying the principles of inclusive pedagogy in initial teacher education: From university-based course to classroom action’, *Revista de Investigacion en Educacion* 11(3), 133–140.

[CIT0045] Swart, E. & Phasha, T.N., 2019, ‘Family and community partnerships’, in E. Landsberg, D. Kruger & E. Swart (eds.), *Addressing barriers to learning: A South African perspective*, pp. 213–234, Van Schaik, Pretoria.

[CIT0046] Topr, D.R., Keane, S.P., Shelton, T.L. & Calkins, S.D., 2010, ‘Parent involvement and student academic performance: A multiple medical analysis’, *Journal of Prevention and Intervention Community* 38(3), 183–197. 10.1080/10852352.2010.486297 PMC3020099 20603757

[CIT0047] Udoba, H.A., 2014, ‘Challenges faced by teachers when teaching learners with developmental disability’, Masters thesis, Dept. of Special Needs Education, University of Oslo.

[CIT0048] UNESCO, 1994, ‘Salamanca statement and framework for action on special needs education’, in World Conference on Special Needs Education: Access and Quality, UNESCO, Ministry of Education and Science in Spain, Salamanca, June 07–10, pp. 1–47.

[CIT0049] UNESCO, 2005, *Guidelines for inclusion: Ensuring access to education for all*, UNESCO, Paris.

[CIT0050] Vaughn, S.R., Bos, S.C. & Schumm, S.J., 2011, *Teaching students who are exceptional, diverse, and at risk in the general education classroom*, 10th edn., Pearson, Hobken, NJ.

[CIT0051] Yoro, A., Fourie, J. & Van Der Merwe, M., 2020, ‘Learning support strategies for learners with neurodevelopmental disorders: Perspectives of recently qualified teachers’, *African Journal of Disability* 9, 561. 10.4102/ajod.vi0.561 32158641 PMC7057733

[CIT0052] Zwane, S.L. & Malale, M.M., 2018, ‘Investigating barriers teachers face in the implementation of inclusive education in high schools in Gege branch, Swaziland’, *African Journal of Disability* 6(7), 391, 10.4102/ajod.v7i0.391 PMC6295749 30568910

